# Automatic selection of resting-state networks with functional magnetic resonance imaging

**DOI:** 10.3389/fnins.2013.00072

**Published:** 2013-05-20

**Authors:** Silvia Francesca Storti, Emanuela Formaggio, Roberta Nordio, Paolo Manganotti, Antonio Fiaschi, Alessandra Bertoldo, Gianna Maria Toffolo

**Affiliations:** ^1^Clinical Neurophysiology and Functional Neuroimaging Unit, Section of Neurology, Department of Neurological, Neuropsychological, Morphological, and Movement Sciences, University HospitalVerona, Italy; ^2^Department of Neurophysiology, Foundation IRCCS San Camillo HospitalVenice, Italy; ^3^Department of Information Engineering, University of PadovaPadova, Italy

**Keywords:** fMRI, BOLD, ICA, resting-state networks, default mode, automatic selection of RSNs

## Abstract

Functional magnetic resonance imaging (fMRI) during a resting-state condition can reveal the co-activation of specific brain regions in distributed networks, called resting-state networks, which are selected by independent component analysis (ICA) of the fMRI data. One of the major difficulties with component analysis is the automatic selection of the ICA features related to brain activity. In this study we describe a method designed to automatically select networks of potential functional relevance, specifically, those regions known to be involved in motor function, visual processing, executive functioning, auditory processing, memory, and the default-mode network. To do this, image analysis was based on probabilistic ICA as implemented in FSL software. After decomposition, the optimal number of components was selected by applying a novel algorithm which takes into account, for each component, Pearson's median coefficient of skewness of the spatial maps generated by FSL, followed by clustering, segmentation, and spectral analysis. To evaluate the performance of the approach, we investigated the resting-state networks in 25 subjects. For each subject, three resting-state scans were obtained with a Siemens Allegra 3 T scanner (NYU data set). Comparison of the visually and the automatically identified neuronal networks showed that the algorithm had high accuracy (first scan: 95%, second scan: 95%, third scan: 93%) and precision (90%, 90%, 84%). The reproducibility of the networks for visual and automatic selection was very close: it was highly consistent in each subject for the default-mode network (≥92%) and the occipital network, which includes the medial visual cortical areas (≥94%), and consistent for the attention network (≥80%), the right and/or left lateralized frontoparietal attention networks, and the temporal-motor network (≥80%). The automatic selection method may be used to detect neural networks and reduce subjectivity in ICA component assessment.

## Introduction

Functional magnetic resonance imaging (fMRI) measures the hemodynamic response induced by neural activity and permits the detection of active brain regions associated with one or more tasks. In a resting-state condition, a state of a spontaneous and endogenous brain activity not intentionally induced externally or voluntarily generated by the subject, fluctuations in the blood oxygenation level-dependent (BOLD) signal reflect the brain's baseline activity (Biswal et al., 1995; Raichle et al., 2001; Greicius et al., 2002; Mulert and Lemieux, 2010). Although the role of these networks is not yet fully understood, their modifications are studied in disease states such as Alzheimer's disease (Greicius et al., 2004). During a task, the most commonly used method to analyze fMRI data is the hypothesis-driven, voxel-based statistical method as a correlation method (Bandettini et al., 1993) and the general linear model (GLM) (Friston et al., 1995). However, because GLM and correlation methods are unable to identify spontaneous brain activity, other techniques are required to identify the spatial patterns of coherent BOLD activity.

The simplest technique currently being developed for the analysis of resting-state data is to extract the BOLD time course from a region of interest (seed region) and then determine the temporal correlation between the extracted signal and the time course from all other brain voxels. A more complex approach is the clustering technique. In the context of resting-state functional connectivity analysis, clustering algorithms have been used to partition the brain into regions (clusters) functionally connected to each other (van den Heuvel et al., 2008). The most commonly employed methods are reviewed in Margulies et al. (2010). Nonetheless, in the absence of a standard paradigm design, a multivariate approach/analysis such as independent component analysis (ICA) is the one most often used.

ICA is an important technique for data-driven analysis as it aims to overcome the problem of blind source separation of signals by dividing the imaging data into several spatial patterns or independent activation maps. With this method, fMRI data can be analyzed in the absence of spatial bounds or a priori knowledge about the activation time courses of the different components, or when a component is activated by specific psychophysiological systems or related to machine noise or other artifacts (McKeown et al., 1998). Furthermore, it has recently been shown that ICA can extract task-related and physiologically-relevant non-task-related components, as well as artifactual components.

Three main methodological issues involve handling of the ICA results. First, each time an analysis is performed, it can induce changes in the estimated independent components (ICs) probably due to either the assumption of statistical independence, which may not hold for the data, or the additive noise that can modify the solution (Vlipaavalniemi and Vigario, 2008). Second, ICA can extract only the number of components defined a priori, and the components are not ranked during decomposition (Boly et al., 2008). This results in overestimation of the dimensionality of the fMRI data for ICA, leading to an excessive number of components with dissociated sources. But the data dimensionality may also be underestimated, rendering the phenomena of interest difficult to separate (Beckmann et al., 2001). While different toolboxes can automatically estimate the number of components, in practice, the dimensions are more often estimated by the user. The third problem is how to identify and isolate the spontaneous networks, so that one can discard non-neuronal noise such as scanner instability, environment noise, head movements, and physiological fluctuation (cardiac and respiratory cycles).

Several methods that order output components have been developed (McKeown, 2000; Formisano et al., 2002; Lu and Rajapakse, 2003; Moritz et al., 2003; De Martino et al., 2007; Schöpf et al., 2010). Lu and Rajapakse order the ICs either according to kurtosis or by incorporating a priori information as constraints; McKeown suggests an hybrid method to separate the task-related components from the artifactual sources, but employing a priori hypothesis to guide the analysis; Formisano and colleagues propose three measures for each IC to solve the selection problem: kurtosis of the component's distribution of the voxel values ranked in descending order, the degree of spatial clustering of its suprathreshold voxels, and one-lag serial correlation of its time course. Finally, algorithm fully exploratory network ICA (FENICA), introduced in the context of ICA group analysis, explores spatially consistent resting-state networks over a group of subjects; it does not require an a priori template definition or visual inspection or single-subject component selection (Schöpf et al., 2010).

Various different approaches to classifying a network across individuals have also been advanced (Calhoun et al., 2001; Greicius et al., 2004; Wang and Peterson, 2008). Greicius and colleagues used the template-matching approach in group-level ICA analysis to detect the default-mode network. Although template matching is an effective means to consistently select analogous networks across individuals, it relies on assuming appropriate templates (Margulies et al., 2010). Calhoun and colleagues addressed the problem of combining components across individuals by entering the individual data sets into a single ICA analysis and then back-reconstructing them. This procedure ensures that the components are consistently ordered across individuals but it is computationally intensive. Another group-level approach (Wang and Peterson, 2008) is based on a clustering algorithm, partner-matching, which automatically identifies the components by clustering them according to robust measures of similarity in their spatial configurations either within or between subjects.

So although ICA decomposition in fMRI is widely used to identify networks, a gold standard selection criterion to select networks with potential functional relevance (i.e., those involved in motor function, visual processing, executive functioning, auditory processing, memory, and the default-mode network) is still lacking.

The aim of this study was to develop a method for automatic selection which could identify the signals representing the networks of interest. Based on a four-step algorithm, the method comprises spatial map filtering, statistical tests and spectral analysis. To test the method, we compared the selection of components on the basis of visual inspection vs. automatic selection in a previously published resting-state fMRI data set of 25 subjects scanned three times on two different occasions (NYU CSC TestRetest, http://www.nitrc.org/projects/nyu_trt/. The first two fMRI resting-state scans were obtained in two scan sessions performed from 5 to 16 months apart, and the third scan about 30 (<45) min after the second one (Shehzad et al., 2009).

## Materials and methods

### Data set and experimental paradigm

Twenty-five participants (mean age, 29.4 ± 8.6 years, 10 males) were scanned three times. The participants had no history of psychiatric or neurological illness, as confirmed by clinical assessment. Informed consent was obtained prior to participation. Data were collected according to protocols approved by the institutional review boards of New York University (NYU) and the NYU School of Medicine (Shehzad et al., 2009; Zuo et al., 2010).

### fMRI data acquisition

For each participant, three resting-state scans were obtained using a Siemens Allegra 3.0 Tesla scanner. Each scan consisted of 197 contiguous EPI functional volumes (*TR* = 2000 ms; *TE* = 25 ms; flip angle = 90°, 39 slices, matrix = 64 × 64; *FOV* = 192 mm; acquisition voxel size = 3 × 3 × 3 mm). Scans 2 and 3 were obtained in a single scan session, 45 min apart, between 5 and 16 months (mean, 11 ± 4 months) after scan 1. All individuals were asked to relax and remain still with their eyes open during the scan. For spatial normalization and localization, a high-resolution T1-weighted magnetization prepared gradient echo sequence was also obtained (MPRAGE, *TR* = 2500 ms; *TE* = 4.35 ms; *TI* = 900 ms; flip angle = 8°; 176 slices, *FOV* = 256 mm) (Shehzad et al., 2009; Zuo et al., 2010).

### Image processing and analysis

#### Pre-processing and PICA decomposition

The functional data were pre-processed using the Multivariate Exploratory Linear Decomposition into Independent Components (MELODIC) version 3.09, part of FSL toolbox (www.fmrib.ox.ac.uk/fsl). The images were smoothed with a Gaussian kernel of full-width at half-maximum of 5 mm but without motion-correction (Bannister et al., 2001). A slice timing correction was used to correct for the different acquisition times. The data were then pre-processed with high-pass temporal filtering (cut-off of 100 s) and with the removal of non-brain structures from the echo planar imaging volumes [Brain Extraction Tool (BET)].

**Figure 1:**
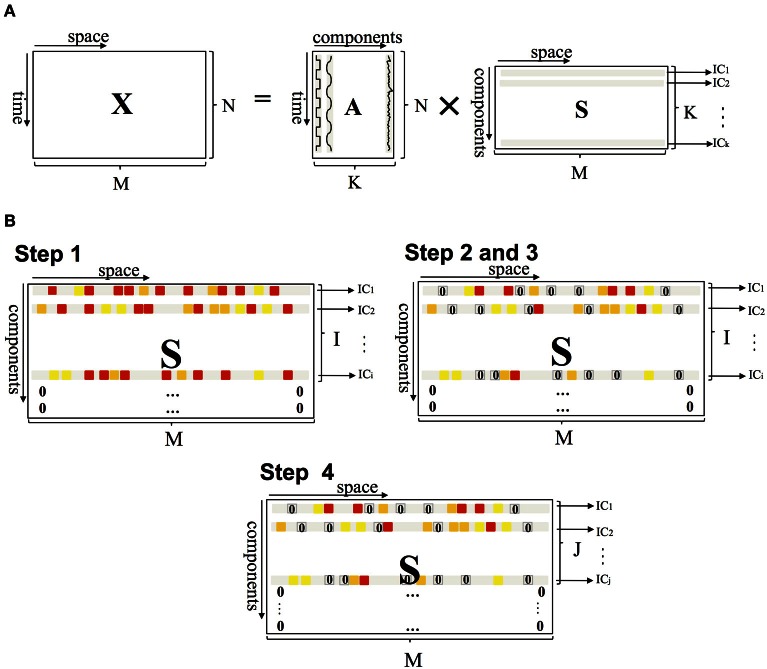
**(A)** PICA of fMRI data. *X* represents a space-time matrix having in its *M* column the *N* dimension time series, the columns of mixing matrix *A* are the time courses, and the rows of matrix *S* are the independent components. Matrix *A* is a rectangular matrix and the number of components *K* is less than the size *N* of data. **(B)** Schematic representation of the method applied to matrix *S.* Steps 1 and 4 operate on the row of matrix *S* (setting some ICs to 0), steps 2 and 3 on the elements (setting some voxels to 0).

The probabilistic PICA method, implemented in FSL software and used in this study, is an extension of the classical spatial ICA framework and estimates sources by maximizing non-Gaussianity in terms of negentropy (Beckmann and Smith, 2004). PICA decomposes a resting-state fMRI dataset into a linear mixture of spatially ICs plus Gaussian noise. The fMRI data set is represented as a space-time matrix *X*, having in its *M* columns the *N*-dimension time series. The relationship between *X* and the IC matrix *S* can be written as:

(1) X = AS + E

where *A* is an *N* × *K* mixing matrix with *K* ≤ *N* (the number *K* of sources is less than the size *N* of data), *S* is a *K* × *M* matrix, and *E* is an *N* × *M* matrix that represents the Gaussian noise (Figure 1A). The mixing matrix *A* is estimated from the data using the maximum likelihood estimation (Beckmann and Smith, 2004); given *A*, the maximum likelihood source estimates (*S*) are obtained using generalized least squares.

The pre-processed data underwent PICA decomposition with the same toolbox. After data reduction by means of principal component analysis (PCA), the number of dimensions (with waveform and spatial maps) for each subject was estimated using the Laplace approximation to the Bayesian evidence of the model order (Minka, 2000; Beckmann and Smith, 2004). The maps were thresholded at a posterior probability threshold of *p* > 0.5 (Beckmann and Smith, 2004). A threshold of *z* scores was then used to visualize the IC maps. Negative *z* scores indicate voxels whose fMRI signals are modulated opposite the IC waveform.

#### A new method for automatically selecting independent components

**Figure 2:**
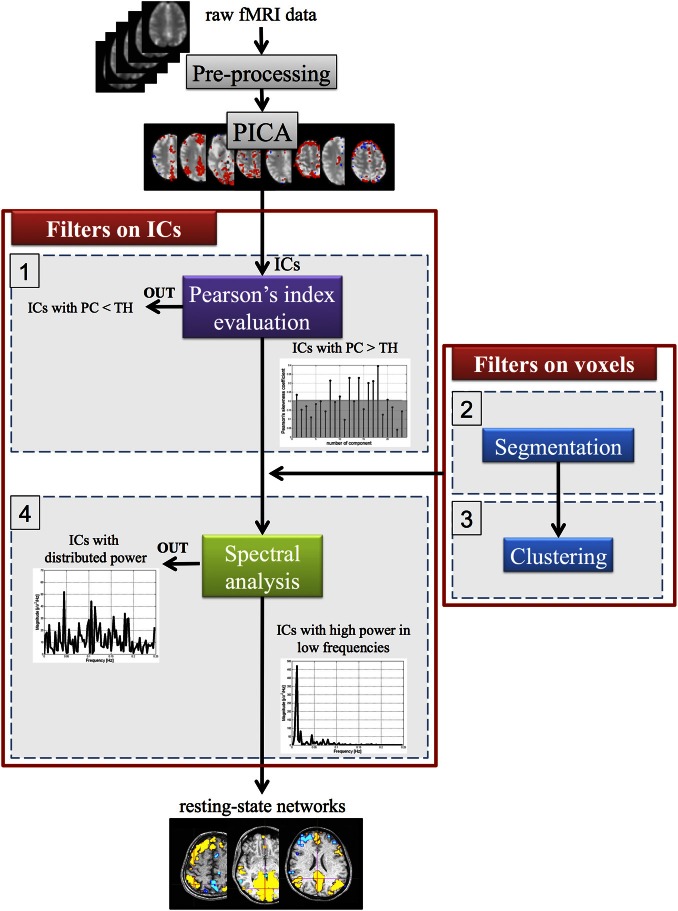
**A schematic representation of the four steps for the automatic selection of ICA components.** TH, threshold value; PC, Pearson's median coefficient of skewness.

Starting from the ICs, as estimated by PICA, a new method was developed to select the optimal number of components related to networks with functional relevance. This algorithm, implemented in Matlab (Mathworks, Sherborn, MA), consists of Pearson's index evaluation and spectral analysis (steps 1–4) to reduce the number of ICs, whereas clustering and segmentation methods (steps 2–3) are used to filter the spatial maps (matrix *S*) at the voxel level. The four steps (schematized in Figure 2) are briefly presented below.

***Pearson's index evaluation (on ICs)***. The method takes into account, for each component, Pearson's index of skewness of each row of the spatial map (matrix *S*) generated by FSL in order to determine whether the data are symmetric or skewed. Given a statistical distribution with measured mean, statistical median, and standard deviation σ, Pearson's median skewness coefficient is:

(2) Pearson's coefficient=3×(mean-median)/σ

**Figure 3:**
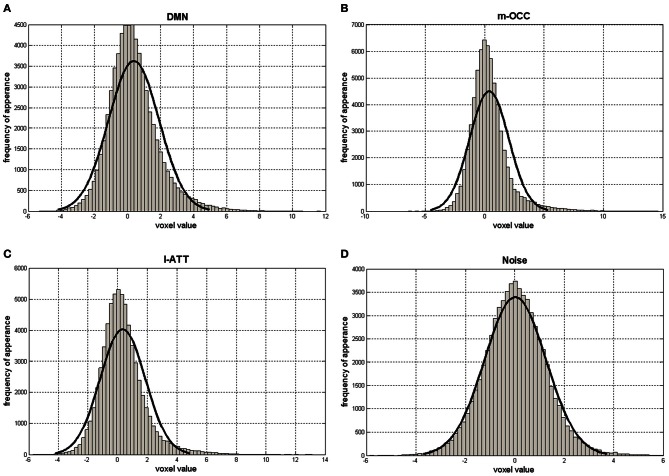
Histograms of *S* matrix rows related to the default-mode network (DMN) **(A)**; the occipital network, the medial visual cortical areas (m-OCC) **(B)**; the attention network, the left lateralized frontoparietal attention networks (l-ATT) **(C)**; and a noisy IC **(D)**. The *x*-axis ranges from negative to positive values. The solid black line represents a Gaussian fit.

Assuming that the noise has a zero-mean Gaussian distribution, the noise-related components are expected to have a Pearson's coefficient close to 0 (Figure 3). The algorithm thus rejects the ICs if Pearson's median coefficient of skewness of the elements in the corresponding row of *S* matrix is lower than a selected threshold (TH), evaluated for each subject as the median of the Pearson's indexes of all elements in the *S* matrix. The ICs and the rows of *S* matrix are then re-ordered so that the rejected ICs are moved to the last positions and set to 0; thus, the matrix *S* has a number of non-zero rows *I* ≤ *K* (Figure 1B).

***Clustering (on map)***. Cluster analysis, implemented by using a *k*-means clustering algorithm (MacQueen, 1967; Golay et al., 1998; Goutte et al., 1999), eliminates those voxels associated with low values for each component. The *k*-means cluster analysis was applied to each non-zero row of *S*, based on the one-dimensional Euclidean distance between activation values. It was performed to divide the candidate of each non-zero row of *S* in *K* clusters. It works on the elements of each non-zero row of *S* (each component selected in step 1), setting to 0 some elements of the *M* columns, i.e., the voxels. After creating the clusters, the algorithm eliminates the voxels belonging to the cluster with the centroid nearest to 0. The centroid with value nearest to 0 represents the voxels in the cluster with the lowest activation values. In order to optimize clustering quality, the silhouette index was used (Kaufman and Rousseeuw, 1990) since it reflects the compactness and separation of clusters. In particular, assuming a specific *i*-th row of *S*, the optimal number of clusters to be used for the values of this selected row, i.e., *K*-value, was determined by using the method proposed in Zhang et al. (2011) based on the evaluation of the silhouette index. For the values of the selected *i*-th row of *S* and for the *j*-th data point, it is defined as follows:

(3) $${\text{SH}}_{j}=(b_{j}-a_{j})/\text{max}(a_{j},b_{j})$$

where *a*_*j*_ is the average Euclidean distance of the *j*-th point to other points in the same cluster and *b*_*j*_ is the average distance of the *j*-th point to points in its nearest neighbor cluster. This index ranges from +1 to −1: a value of unity indicates that the point is very distant from its neighbor clusters, whereas a value of −1 indicates that the *j*-th point is closer to points in its nearest neighbor cluster than to those in its own cluster. The number of clusters was determined by maximizing the average silhouette value over a range of *k*, since a larger average silhouette indicates better clustering quality (Zhang et al., 2011).

***Segmentation (on map)***. PICA decomposition models the signal also in subcortical structures since the *S* matrix includes signals from white matter and ventricles. To minimize pulsation effects from the cerebrospinal fluid and restrict the activations to the gray matter, the fMRI data were segmented with Statistical Parametric Mapping (SPM) version 8 (http://www.fil.ion.ucl.ac.uk/spm/). The voxels in each component with at least a 90% probability of belonging to the white matter or cerebrospinal fluid were cancelled (Keihaninejad et al., 2010; Polanía et al., 2012).

***Spectral analysis (on ICs)***. For each component *i*, selected in step 1, the voxels s_*i*, *m*_ selected in step 3 were identified. The relative fMRI time courses *x*_*n*, *m*_ = *a*_*n*, *i*_ · *s*_*i*, *m*_, with *m* = 1 … *M* and *n* = 1 … *N* were baseline corrected, detrended and averaged. For each component, the fast Fourier transform (FFT) (using the periodogram method) was then applied to the mean fMRI time course, and the relative power was estimated in the three bands of interest: *P*_1_ [0 − *f*_1_ Hz], *P*_2_ [*f*_1_ − *f*_2_ Hz] and *P*_3_ [> *f*_2_ Hz], according to Equation 4:

(4) $$P_{1}=\frac{\int_{0}^{f_{1}}P_{x}(f)}{\int_{0}^{a}P_{x}(f)}df,P_{2}=\frac{\int_{f_{1}}^{f_{2}}P_{x}(f)}{\int_{0}^{a}P_{x}(f)}df,P_{3}=\frac{\int_{f_{2}}^{a}P_{x}(f)}{\int_{0}^{a}P_{x}(f)}df$$

where *f*_1_ = 0.01 Hz and *f*_2_ = 0.1 Hz. *P*_x_(*f*) (μV^2^/Hz) is the power spectral density and *a* depends on acquisition parameters. Since resting-state networks are characterized by slow fluctuations of functional imaging signals between 0.01 and 0.1 Hz (*P*_2_) (Cordes et al., 2000; Damoiseaux et al., 2006; De Martino et al., 2007; Mantini et al., 2007), the components with *P*_2_ < 50% and with *P*_1_+ *P*_2_ < 90% were rejected. After spectral analysis, matrix *S* has *J* ≤ *I* columns (i.e., components) (Figure 1B). Because intrinsic connectivity is detected in the very low-frequency range (Cordes et al., 2001), also other researchers have applied a frequency filter to remove any components in which a high-frequency signal (>0.1 Hz) constituted 50% or more of the power in the Fourier spectrum (Greicius et al., 2004). In keeping with this hypothesis, we applied those thresholds.

#### Assessment

To quantify the method's ability to select meaningful ICA components, the resting-state networks identified by the automatic method were compared against those visually identified based on accuracy and precision defined as:

(5) Accuracy=(TP+TN)/(TP+TN+FP+FN)

(6) Precision=TP/(TP+FP)

where:

- true positives (TP) are the number of resting-state networks identified by an expert and correctly recovered by the automatic method,
- false positives (FP) are the number of false resting-state networks,
- false negatives (FN) are the number of missed resting-state networks,
- true negatives (TN) are the components correctly rejected by the automatic method.

Careful attention was paid to the selection of the resting-state networks. Visual selection for each subject entailed visual inspection by two experts (a neurophysiologist and an bioengineer expert in neuroscience) who recognized the components independently. All networks were evaluated and compared with those reported in the literature. Few disagreements were discussed within the group and resolved by reference to a third author that assessed those components. The networks listed in **Tables S1**, **S2** reflect this final result, however, the components discussed within the group were highlighted. Two different situations can occur: (a) the IC, evaluated discordantly by the two evaluators, is accepted after discussion within the group (^*^) and (b) the IC, evaluated discordantly by the two evaluators, is rejected (§). Twelve components were accepted after discussion within the group (5 in the first scan, 3 in the second scan and 2 in the third scan). In the first scan 3 components were rejected after discussion. The networks detected on the visual inspection have potential functional relevance and consist of regions known to be involved in motor function, visual processing, executive functioning, auditory processing, memory, and the default-mode network. Eight of the most common and consistent resting-state networks were visually identified: the default-mode network; the attention networks (right and left lateralized frontoparietal attention networks); the executive-control network; the occipital networks (medial and lateral visual cortical areas); the temporal network; and the sensorimotor network (Cole et al., 2010; Figure 1). The components were defined on the basis of networks well known in the literature and anatomical information.

The reproducibility (e.g., how many components would be consistent when the resting scan is repeated in the same subject) was also tested for the components detected by visual selection and by automatic selection. The first and the second fMRI resting-state scans were compared to test the reproducibility of the networks in the same subject after a period of several months had elapsed, whereas the second and the third scans were compared to test the reproducibility on the same day.

## Results

**Tables S1**, **S2** summarize the performance of our method in 25 subjects (three recordings per subject): in the first scan 194 out of a total of 577 components decomposed by PICA were true positives, i.e., the number of resting-state networks the method correctly recovered; 22 were false positives; 7 were false negatives; and 354 were true negatives with an accuracy of 95% and a precision of 90%. In the second scan 191 out of a total of 506 components selected by FSL were true positives; 21 were false positives; 2 were false negatives; and 292 were true negatives with an accuracy of 95% and a precision of 90%. In the third scan 182 out of a total of 533 components selected by FSL were true positives; 34 were false positives; 5 were false negatives; and 312 were true negatives with an accuracy of 93% and a precision of 84%. **Tables S1**, **S2** underline that in 27 out of 75 recordings the algorithm fully detected the visually identified networks. The automatic method missed only 14 of the visually chosen components. In several other cases, it also selected additional components not identified as neuronal activations. More important for the recognition of resting-state networks, however, is the number of false negatives.

Although the number of components selected by FSL and then provided to the algorithm varied widely (range, 16–53 components) (**Tables S1**, **S2**), the algorithm performed similarly.

**Figure 4:**
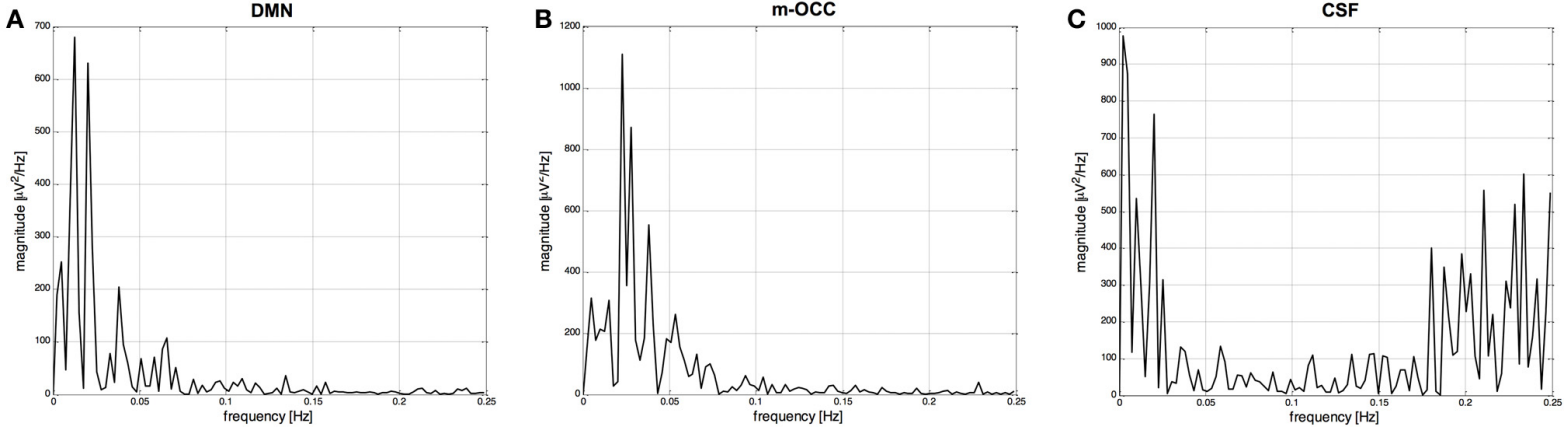
**(A)** and **(B)** Power spectral densities (μV^2^/Hz) of two IC time courses selected by the algorithm: the default-mode network (DMN) and occipital network, the medial visual cortical areas (m-OCC), after clustering and segmentation. **(C)** Power spectral density of an IC time course related to cerebrospinal fluid (CSF) and rejected by the spectral analysis.

The frequency with the largest amplitude in *P*_2_ was identified by spectral analysis as the dominant frequency for all resting-state networks. This is exemplified in Figure 4, which shows for one subject the power spectra of two IC time courses selected by the algorithm (default-mode network and occipital network) after clustering and segmentation, as well as the power spectrum of an IC time course related to cerebrospinal fluid and rejected by the spectral analysis. Because of the wide variability of resting-state network time courses, it was difficult to make an objective evaluation in the time domain. When we applied the FFT to the time course of the fMRI data, we observed recognizable peaks at low frequencies for the resting-state networks, with the highest percentage of power contained in *P*_2_.

**Figure 5:**
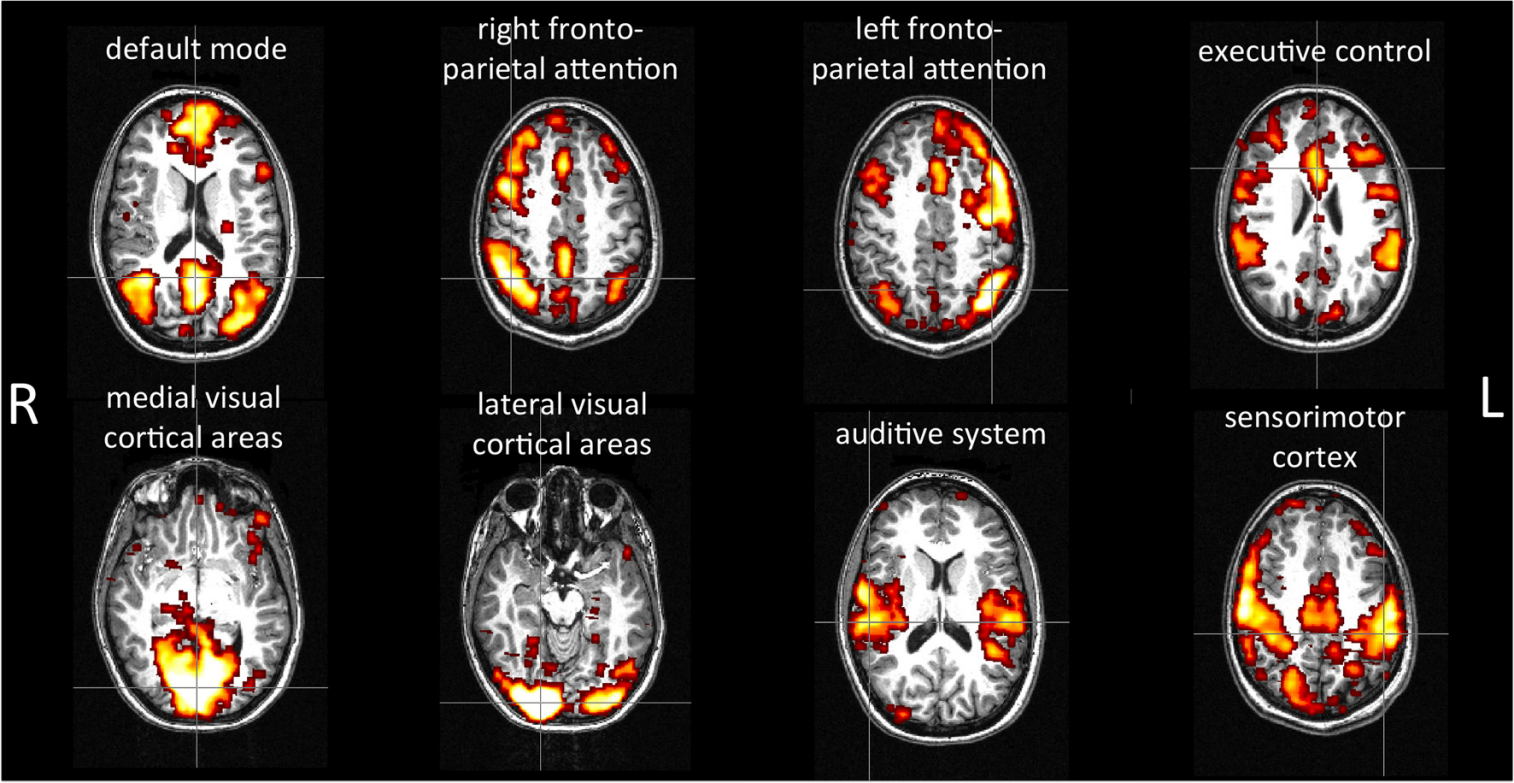
**Example of resting-state networks detected by the method in a single subject.** The anatomical and functional data were registered by using affine registration on fMRIB's Linear Image Registration Tool (FLIRT), using the anatomical images as the reference. The images were visualized using the FSLView toolbox.

Figure 5 shows an example of selected networks in a single subject: the default-mode network, a baseline activity that is suspended during specific goal-directed behaviors; the attention networks (right and left lateralized frontoparietal attention networks) implicated in working memory and cognitive attentional processes; the executive-control network involved in planning, decision making, and error detection; the occipital networks (medial and lateral visual cortical areas) associated with visual processing; the temporal network; and the sensorimotor network.

Summing up, the default-mode network was present on almost all recordings but one (scan 1 sub47000); the occipital network, which includes the medial visual cortical areas, was present in all subjects and scans. Also the other networks were highly present as described in Tables 1, **S1**, **S2**. At this level of decomposition, the sensorimotor IC included also auditory areas in the majority of the subjects.

**Table 1:** **Subjects nos. 1–25, NYU data set**.

**RSN**	**PRESENCE IN**			**VISUAL**		**AUTOMATIC**	
	**1**	**2**	**3**	**1 vs. 2**	**2 vs. 3**	**1 vs. 2**	**2 vs. 3**
DMN	96%	100%	100%	96%	100%	92%	98%
m-OCC	100%	100%	100%	100%	100%	96%	94%
l-OCC	56%	80%	64%	44%	56%	44%	56%
ATT	96%	88%	96%	84%	84%	80%	82%
EXC	64%	56%	48%	40%	28%	38%	24%
TEMP-MOT	96%	88%	88%	84%	76%	80%	72%

*Presence of resting-state networks in the three scans and measure of reproducibility of the visual selection (VISUAL) and the automatic selection (AUTOMATIC). For each network, the visual and method reproducibility are the percentage of subjects in which the network has been identified in the two considered recordings by visual and automatic selection, respectively. Recordings 1 vs. 2 and 2 vs. 3 are compared*.

*RSN, resting-state networks; DMN, default-mode network; m-OCC, medial visual cortical areas; l-OCC, lateral visual cortical areas; ATT, right-left lateralized frontoparietal attention networks; EXC, executive-control network; TEMP-MOT, auditive system-sensorimotor cortex*.

As detailed in Table 1, the reproducibility of the networks by means of visual and automatic selection was very close, although the latter was somewhat smaller. The reproducibility was highly consistent in each subject for the default-mode network (≥92%) and for the occipital network, which includes the medial visual cortical areas, (≥94%), and it was consistent for the attention network (≥80%), the right and/or left lateralized frontoparietal attention networks, and the temporal-motor network (≥80%).

## Discussion

The aim of the method described here is to provide for the automatic selection of ICA components, which is usually done visually, thus eliminating the subjectivity in component selection while reducing the time needed to analyze fMRI data. The method integrates time, frequency and spatial processing within an automatic selection procedure that is reliable, flexible and widely applicable. When applied to the NYU data set, consisting of fMRI images of 25 healthy subjects, the algorithm demonstrated high accuracy (first scan: 95%, second scan: 95%, third scan: 93%) and precision (first scan: 90%, second scan: 90%, third scan: 84%), confirming its ability to automatically detect the visually identified networks. Our results are in line with previous studies which have demonstrated the presence of well-known resting-state networks. The reproducibility of the default-mode network, the occipital network, which includes the medial visual cortical areas, the attention network, the right and/or left lateralized frontoparietal attention networks, and the temporal-motor network is high; nevertheless, it depends on the reproducibility of the networks separated at ICA decomposition. An elevated number of false positives is less critical than an elevated number of false negatives. In fact, an higher number of false negatives, i.e., the method is not able to detect components visually identified as resting networks, implies a loss of useful information that cannot be recovered. Differently, a high number of the false positives, i.e., additional components not visually identified as neuronal activations, implies components that had to be re-evaluated by the user. Actually, the number of false negatives, i.e., the number of missed resting-state networks, was well controlled: the automatic method missed only 14 of the visually chosen components. Conversely, a higher number of false positives were detected.

In this method, the first step is to evaluate Pearson's median coefficient of skewness of each row of the spatial map. This serves to reject the components that show an index close to 0, i.e., those related to the noisy components in agreement with the model specifications of additive Gaussian noise presence. In the *K*-means clustering and segmentation steps, some specific voxels are set to 0. The iterative partitioning of *K*-means minimizes the sum, over all clusters, of the within-cluster sums of point-to-cluster-centroid distances. The importance of the clustering is that it groups voxels with similar characteristics and, as result, the active clusters are concentrated without weak and isolated activations. Also the white matter and cerebrospinal fluid segmentations are an important step in the analysis to determine the activations only on the cortical surface of the brain and improve the method's accuracy. Other solutions can be hypothesized: remove components with main representation in the white matter or cerebrospinal fluid voxels or apply the segmentation before PICA, limiting the activation in the gray matter. These alternatives could be considered to possibly improve accuracy and precision of the method. Both clustering and segmentation work at voxel level improving the signal-to noise ratio of the component mean time course.

Consequently, the spectral analysis was applied only to voxels selected by previous steps. In the frequency analysis step, the FFT (Kiviniemi et al., 2000), the cross-correlation analysis with the prior definition of a reference region (Biswal et al., 1995), the frequency analysis of cross-correlations (Cordes et al., 2001), the coherence analysis with a user-defined seed region (Sun et al., 2004), and spectral coherence analysis without seed regions (Thirion et al., 2006) used in this field showed that the waveforms of these spontaneous activity patterns have a prominently low-frequency contribution and that only the frequencies less than 0.1 Hz contribute significantly to interregional functional connectivity (Cordes et al., 2000; Laufs et al., 2003; Damoiseaux et al., 2006; De Martino et al., 2007; Mantini et al., 2007).

In order to discriminate the resting-state networks from physiological fluctuation, Soldati et al. (2009) recently applied spectral analysis to group-ICA time courses using a statistical approach to determine if the two classes were statistically different. Differently, we investigated the power spectra of the ICs to identify the power concentrated in the range of interest (0.01–0.1 Hz) and found high power in the low-frequency range for the resting-state networks, as typically observed in the literature.

In MELODIC, automatic estimation of the number of spatial maps for each subject is useful when one does not want to subdivide the networks into different components (Beckmann and Smith, 2004). At low dimensionalities, the signal sources merge into singular components, whereas high orders of decomposition can allow for detailed evaluation of resting-state networks. We noted, however that increasing the dimensionality further reduced reliability. The choice of the dimensionality in ICA is a critical point, but the automatic estimation of FSL software provides a tradeoff between the two solutions. Networks, such as the default mode network, the attention network and the motor-temporal network, split into multiple components on more than half of the recordings, in which they were detected; whereas other networks, such as the occipital networks and the executive-control network, below the 25%. Nevertheless, the method of selection can be used without this pre-selection, i.e., on a number of ICs equal to the number of time points in the data.

For the physiological classification of the networks related to neuronal activity, the BOLD networks may be classified as unconstrained behavior or mental activities similar to modulations induced by external stimuli and as intrinsic activity similar to the anatomy. The origin of these networks is still an open question. It is unclear whether the fluctuations are independent of neuronal function or reflect neuronal baseline activity without task (Damoiseaux et al., 2006). Our study confirms the presence of various networks distinguishes as: the default-mode network, the attention network; the occipital network; and the motor-temporal network.

In 2001 Marcus Raichle and colleagues coined the term “default-mode” to describe a resting-state brain function demonstrating the existence of an organized, baseline default mode of brain function that is present as a baseline or default state and is suspended during specific goal-directed behaviors (Raichle et al., 2001). This network has been identified in both animals (Lu et al., 2007; Vincent et al., 2007) and humans. In a positron-emission tomography (PET) and fMRI study, the default-mode network showed a consistent decrease from a relative baseline during a specific goal-directed behavioral task (Raichle et al., 2001). The default-mode network closely resembles the brain areas found to be involved in random episodic silent thinking and it is putatively associated with internal processing (Andreasen et al., 1995). Also, the network sustains its activity despite decreasing vigilance.

The default-mode network component includes the posterior cingulated cortex/precuneous, the medial prefrontal cortex, and the bilateral temporoparietal junctions. Two studies (Laufs et al., 2003; Mantini et al., 2007) described a bilateral frontoparietal pattern, including the intraparietal sulcus and frontal eye field, a network mediating goal-directed stimulus-response selection associated with alpha desynchronization, which is probably due to unconstrained behavior or a metal activity of which a subject is consciously aware (Laufs et al., 2003; Mantini et al., 2007). Recent studies on functional connectivity have demonstrated the presence of differentiation inside the default-mode network, linking the precuneus (a site with the highest degree of interactions) and posterior cingulated cortex with visual-spatial and attention networks and the medial prefrontal cortex with the motor control circuit (Uddin et al., 2008). Fox and colleagues found activations in two attentional systems that persist also in the absence of external stimuli: a bilateral dorsal attention system usually involved in top–down orienting of attention and a right-lateralized ventral attention system which reorients attention in response to salient sensory stimuli. Many hypotheses can be advanced regarding spontaneous activity and its similarities to task-evocated activity. What might serve is to organize neuronal activity conceived of as either a memory of previous use or a prediction regarding future use (Fox et al., 2006).

Finally, a network dedicated to visual processing (the occipital networks) involving the retinotopic occipital cortex and the temporal-occipital regions have been identified, and the BOLD signal fluctuations associated with it may be correlated with the electroencephalographic power variations of alpha (Mantini et al., 2007).

In conclusion, although several previous studies used data-driven techniques to assess functional activity during the resting-state condition, they did not develop a selection criterion to separate the ICs related to neural networks from those related to noise. With the algorithm described here, these “rhythms” can be identified and an adequate number of activation maps obtained with high accuracy and precision. This time-saving method allows to automate the selection of components related to resting-state networks, and reducing subjectivity in the classification of the ICs. Furthermore, the method increases the repeatability of resting-state network selection, making it particularly useful in multicenter studies or in studies where a major concern is to minimize the variability in results due to the subjectivity of visual selection.

### Conflict of interest statement

The authors declare that the research was conducted in the absence of any commercial or financial relationships that could be construed as a potential conflict of interest.
