# Supplementary material for: Automatic selection of resting-state networks with functional magnetic resonance imaging
Source: Front Neurosci. 2013 May 20;7:72. doi: 10.3389/fnins.2013.00072 (PMC3657627; doi:10.3389/fnins.2013.00072)
Supplement: Supplementary file 1 [file DataSheet1.PDF]

Supplementary material

Table S1. Subjects nos. 1–13. Comparison between automatic and visual selection of independent components. "S" refers to the number of the subject, "ICs" to the number of automatic components estimated by the FSL software, "M" to the components selected by the automatic method.

NYU_TRT_sessiona

S	ICs	Visual Selection	M	ICs	Visual Selection	M	ICs	Visual Selection	M
sub05676	19	2 (m-OCC)	2	18	4 (m-OCC)	4	19	8 (l-OCC)	8
		6 (DMN)	NF		6 (r-l-ATT)	6		10 (m-OCC)	10
		7 (TEMP-MOT)	7		8 (r-l-ATT)	8		11 (EXC)	11
		8 (DMN)	8		9 (DMN)	9		13 (DMN)	13
		10 (EXC)	NF		NF	10		14 (r-ATT)	14
		12 (r-l-ATT)	12		12 (DMN)	12		15 (l-ATT)	15
		19 (EXC)	19		14 (EXC)	14		17 (TEMP-MOT)	NF
sub08224	23	NF	1	17	5 (r-l-ATT)	5	22	2 (m-OCC)	2
		8 (DMN)	8		6 (DMN)	6		6 (TEMP-MOT)	6
		12 (TEMP-MOT)	NF		7 (TEMP-MOT)	7		7 (l-OCC)	7
		14 (l-ATT)	14		8 (l-OCC)	8		8 (MOT)	8
		16 (DMN)	16		10 (m-OCC)	10		10 (TEMP)	10
		17 (r-ATT)	17		15 (DMN)	15		11 (r-l-ATT)	11
		18 (m-OCC)	18		16 (l-OCC)	16		13 (EXC)	13
								14 (m-OCC)	NF
								15 (l-OCC)	15
		21 §						16 (DMN)	16
sub08889	19	5 (l-OCC)	5	18	NF	1	21	3 (r-ATT)	3
		6 (EXC)	6		5 (DMN)	5		4 (DMN)	4
		8 (TEMP-MOT)	8		8 (TEMP-MOT)	8		NF	5
		9 (DMN)	9		13 (r-l-ATT)	13		6 (TEMP-MOT)	6
		11 (m-OCC)	11		14 (EXC)	14		8 (l-ATT)	8
		12 (r-l-ATT)	12		15 (m-OCC)	15		9 (m-OCC)	9
					NF	18		11 (MOT)	11
								16 (TEMP-MOT)	16
								NF	21
sub09607	19	1 (TEMP-MOT)	1	18	4 (m-OCC)	4	18	5 (l-OCC)	5
		3 (DMN)	3		5 (m-OCC)	5		9 (m-OCC)	9
		9 (DMN)	9		6 (TEMP-MOT)	6		10 (DMN)	10
		10 (r-l-ATT)	10		11 (l-OCC)	11		NF	13
		11 (r-l-ATT)	11		12 (DMN)	12		14 (TEMP-MOT)	NF
		12 (m-OCC)	12		15 (l-ATT)	15		15 (r-l-ATT)	15
		16 (r-ATT)	16		16 (r-l-ATT)	16		16 (r-l-ATT)	16
					18 (DMN)	NF			
sub14864	21	1 (TEMP-MOT)	1	18	NF	1	19	1 (TEMP-MOT)	1
		2 (m-OCC)	2		5 (DMN)	5		2 (TEMP-MOT)	2
		5 (l-OCC)	5		6 (EXC)	6		3 (DMN)	3
		9 (l-OCC)	9		7 (l-ATT)	7		4 (l-OCC)	4
		10 (m-OCC)	10		8 (r-l-ATT)	8		5 (r-l-ATT)	5
		11 (l-ATT)	11		10 (DMN)	10		9 (l-OCC)	9
		14 (EXC) *	14		11 (l-OCC)	11		10 (r-l-ATT)	10
		15 (r-ATT)	15		13 (m-OCC)	13		12 (m-OCC)	12
		17 (DMN)	17		14 (r-ATT)	14		13 (TEMP)	13
					15 (MOT)	15		14 (EXC)	14
								15 (DMN)	15

S	ICs	Visual Selection	M	ICs	Visual Selection	M	ICs	Visual Selection	M
sub18604	24	8 (r-ATT)	8	28	NF	1	26	NF	2
		10 (r-l-ATT)	10		4 (FRO)	4		NF	4
		11 (l-OCC)	11		9 (TEMP-MOT)	9		12 (r-l-ATT)	12
		12 (m-OCC)	12		10 (l-OCC)	10		13 (DMN)	13
		13 (TEMP-MOT)	13		11 (DMN)	11		15 (DMN)	15
		15 (DMN)	15		18 (m-OCC)	18		16 (m-OCC)	16
		17 (l-ATT)	17		19 (EXC)	19		NF	17
		18 (DMN)	18		NF	21		20 (TEMP-MOT)	20
					22 (r-l-ATT)	22		21 (l-ATT)	21
					23 (EXC)	23		23 (r-ATT)	23
					24 (DMN)	24			
					27 (l-ATT)	27			
sub22894	27	NF	3	23	2 (FRO)	2	24	NF	11
		7 (TEMP-MOT)	7		11 (m-OCC)	11		14 (m-OCC)	14
		14 (r-l-ATT)	14		14 (m-OCC)	14		16 (TEMP)	16
		15 (m-OCC)	15		19 (DMN)	19		17 (l-OCC)	17
		21 (DMN)	NF		21 (MOT)	21		20 (DMN)	20
		24 (DMN)	24		23 (l-OCC)	23		21 (r-l-ATT)	21
								23 (l-OCC)	23
		25 §							
sub27641	53	3 (MOT)	3	23	NF	2	25	7 (TEMP-MOT)	7
		NF	7		4 (l-OCC)	4		12 (m-OCC)	12
		NF	10		6 (EXC)	6		14 (DMN)	14
		12 (m-OCC)	12		8 (TEMP-MOT)	8		NF	21
		13 (m-OCC)	13		10 (r-l-ATT)	10			
		14 (l-OCC)	14		14 (m-OCC)	14			
		16 (DMN)	16		15 (r-l-ATT)	15			
		18 (l-OCC)	18		17 (DMN)	17			
		19 (MOT)	19		19 (DMN)	19			
		20 (MOT)	20						
		21 (r-l-ATT)	21						
		22 (r-l-ATT) *	22						
		NF	25						
		27 (DMN)	27						
		29 (r-ATT)	29						
		31 (DMN) *	31						
		42 (EXC)	42						
		44 (MOT)	44						
		NF	50						
		51 (l-OCC)	51						
sub33259	22	4 (TEMP-MOT)	4	18	6 (m-OCC)	6	19	NF	2
		5 (m-OCC)	5		8 (DMN)	8		3 (TEMP-MOT)	3
		7 (DMN)	7		9 (EXC)	9		5 (m-OCC)	5
		11 (EXC)	11		12 (m-OCC)	12		NF	8
		14 (l-OCC)	14		NF	17		9 (l-OCC)	9
		18 (r-l-ATT)	18					10 (r-l-ATT)	10
		NF	19					NF	12
		21(r-ATT)	21					15 (DMN)	15

S	ICs	Visual Selection	M	ICs	Visual Selection	M	ICs	Visual Selection	M
sub34482	19	1 (TEMP-MOT)	1	22	NF	1	24	1 (FRO)	1
		3 (r-l-ATT)	3		2 (FRO)	2		2 (TEMP)	2
		4 (m-OCC)	4		3 (l-OCC)	3		3 (MOT)	3
		5 (FRO)	5		4 (EXC)	4		4 (r-l-ATT)	4
		6 (EXC)	6		5 (TEMP)	5		NF	5
		NF	8		8 (DMN)	8		NF	6
		10 (DMN)	10		12 (l-ATT)	12		8 (l-ATT)	8
		12 (DMN)	12		13 (r-ATT)	13		9 (DMN)	9
		13 (r-l-ATT)	13		14 (TEMP-MOT)	14		11 (m-OCC)	11
					17 (m-OCC)	17		15 (r-ATT)	15
								18 (EXC)	18
								19 (DMN)	19
								20 (TEMP-MOT)	20
sub36678	19	4 (DMN)	4	18	2 (DMN)	2	21	2 (DMN)	2
		5 (TEMP-MOT)	5		3 (l-OCC)	3		5 (r-l-ATT)	5
		6 (DMN)	6		4 (DMN) *	4		6 (m-OCC)	6
		7 (l-OCC)	7		5 (m-OCC)	5		10 (EXC)	10
		8 (EXC)	8		NF	7		11 (MOT)	11
		10 (m-OCC)	10		9 (r-l-ATT)	9		13 (l-ATT)	13
		12 (l-ATT)	12		10 (r-ATT)	10		14 (r-ATT)	14
		13 (r-l-ATT)	13		12 (DMN)	12			
		14 (MOT)	14		13 (TEMP-MOT)	13			
					14 (l-ATT)	14			
sub38579	22	5 (r-ATT)	5	20	NF	2	24	5 (TEMP-MOT)	5
		7 (m-OCC)	7		5 (l-OCC)	5		8 (r-l-ATT)	8
		12 (DMN)	12		6 (DMN)	6		9 (DMN)	9
		13 (TEMP-MOT)	13		7 (r-ATT)	7		NF	11
		16 (l-ATT)	16		11 (m-OCC)	11		12 (l-OCC)	12
		19 (DMN)	NF		19 (l-ATT)	19		16 (m-OCC)	16
								20 (EXC)	20
								22 (l-ATT)	22
sub39529	22	1 (r-l-ATT)	1	20	4 (TEMP-MOT)	4	19	2 (MOT)	2
		2 (TEMP-MOT)	2		6 (r-l-ATT)	6		3 (l-OCC)	3
		3 (FRO)	3		7 (DMN)	7		NF	4
		4 (r-l-ATT)	4		8 (l-OCC)	8		6 (TEMP-MOT)	6
		5 (DMN)	5		9 (TEMP-MOT)	9		8 (r-l-ATT)	8
		7 (m-OCC)	7		13 (r-l-ATT)	13		NF	9
		8 (l-ATT)	8		16 (m-OCC)	16		10 (DMN)	10
		9 (DMN)	9		19 (DMN)	19		15 (m-OCC)	15
		12 (EXC)	12						

DMN = default-mode network, m-OCC (medial visual cortical areas), l-OCC (lateral visual cortical areas), r-ATT (right lateralized frontoparietal attention networks), l-ATT (left lateralized frontoparietal attention networks), EXC (executive-control network), FRO (frontal network), TEMP (auditive system), MOT (sensirimotor cortex), TEMP-MOT (auditive system–sensorimotor cortex), NF = not found. **(*) the IC, evaluated discordantly by the two evaluators, is accepted after discussion within the group, (§) the IC, evaluated discordantly by the two evaluators, is rejected.**

Table S2. Subjects nos. 14–25. Comparison between the automatic and visual selection of independent components. "S" refers to the number of the subject, "ICs" to the number of automatic components estimated by the FSL software, "M" to the components selected by the automatic method.

NYU_TRT_sessionb

S	ICs	Visual Selection	M	ICs	Visual Selection	M	ICs	Visual Selection	M
sub45463	20	NF	1	17	3 (r-l-ATT)	3	22	4 (DMN)	4
		11 (r-l-ATT)	11		7 (DMN)	7		5 (r-ATT)	5
		12 (DMN)	12		8 (m-OCC)	8		7 (r-l-ATT)	7
		13 (l-OCC)	13		10 (l-OCC)	10		8 (m-OCC)	8
		14 (m-OCC)	14		13 (TEMP-MOT)	13		13 (DMN)	13
		15 (EXC)	15					14 (l-OCC)	14
		17 (DMN)	17					18 (r-ATT)	18
		18 (TEMP-MOT)	18						
		19 (l-OCC)	19						
sub47000	16	3 (TEMP-MOT)	3	22	NF	6	25	NF	1
		5 (m-OCC)	5		7 (DMN)	7		NF	2
		8 (l-OCC)	8		8 (TEMP-MOT) *	8		3 (FRO)	3
		13 (EXC)	13		10 (DMN)	10		7 (m-OCC)	7
					11 (m-OCC)	11		12 (r-l-ATT)	12
					12 (DMN)	12		15 (DMN)	15
					15 (r-ATT)	15		16 (l-ATT)	16
					16 (l-ATT)	16		19 (DMN)	19
					17 (r-l-ATT)	17		23 (r-ATT)	23
		16 §			18 (TEMP-MOT)	18			
sub49401	30	1 (FRO)	1	28	NF	1	29	NF	2
		NF	2		NF	2		NF	3
		4 (l-ATT)	4		6 (l-OCC)	6		NF	6
		10 (m-OCC)	10		NF	8		7(DMN)	7
		12 (DMN)	12		9 (DMN)	9		NF	8
		NF	13		10 (r-l-ATT)	10		9 (l-OCC)	9
		14 (l-OCC)	14		11 (TEMP)	11		NF	10
		15 (TEMP-MOT)	15		12 (m-OCC)	12		11 (m-OCC)	11
		16 (EXC)	16		13 (EXC)	13		NF	12
		17 (TEMP)	17		14 (DMN)	14		13 (r-ATT)	13
		NF	18		18 (FRO)	18		14 (TEMP)	14
		19 (TEMP)	19		NF	19		16 (TEMP-MOT)	16
		20 (r-l-ATT)	20		20 (r-ATT)	20		19 (MOT) *	19
		NF	22		22 (l-OCC)	22		21 (DMN)	21
		23 (MOT)	23		23 (TEMP-MOT)	23		22 (r-l-ATT)	22
		26 (DMN)	26					23 (DMN)	23
								NF	28
sub52738	22	4 (DMN)	4	17	3 (l-OCC)	3	17	1 (FRO)	1
		6 (l-ATT)	6		6 (m-OCC)	6		6 (l-ATT)	6
		7 (FRO)	7		8 (m-OCC)	8		7 (m-OCC)	7
		9 (l-OCC)	9		9 (DMN)	9		8 (r-ATT)	8
		11 (m-OCC)	11		10 (TEMP-MOT)	10		9 (TEMP-MOT)	9
		12 (DMN)	12		11 (EXC)	11		10 (l-OCC)	10
		16 (MOT)	16		12 (DMN)	12		11 (DMN)	11
		18 (EXC)	18					14 (DMN)	14
		19 (TEMP-MOT)	19						
		20 (m-OCC)	20						

S	ICs	Visual Selection	M	ICs	Visual Selection	M	ICs	Visual Selection	M
sub55441	22	NF	2	19	4 (l-OCC)	4	21	NF	2
		4 (TEMP-MOT)	4		5 (DMN)	5		7 (m-OCC)	7
		6 (DMN)	6		7 (TEMP-MOT)	7		9 (m-OCC)	9
		7 (m-OCC)	7		8 (m-OCC)	8		10 (TEMP-MOT)	10
		8 (EXC)	8		9 (r-l-ATT)	9		13 (EXC)	13
		13 (l-OCC)	13		11 (DMN)	11		14 (DMN)	14
		14 (DMN)	14		12 (m-OCC)	12		15 (l-OCC)	15
		15 (DMN)	15		14 (r-ATT)	14		16 (r-ATT)	16
		16 (r-l-ATT)	NF		16 (l-OCC)	16		18 (DMN)	18
		NF	20		17 (l-ATT)	17		19 (l-ATT)	19
								20 (r-l-ATT)	20
sub58949	26	1 (FRO)	1	23	3 (m-OCC)	3	21	NF	2
		NF	3		5 (r-l-ATT)	5		3 (m-OCC)	3
		6 (TEMP-MOT)	6		8 (TEMP-MOT)	8		5 (TEMP-MOT)	5
		7 (m-OCC)	7		9 (r-l-ATT) *	9		7 (DMN)	7
		8 (l-OCC)	8		10 (DMN)	10		8 (r-ATT)	8
		9 (DMN)	9		15 (l-OCC)	15		9 (l-ATT)	9
		10 (r-l-ATT)	10		17 (EXC)	17		NF	13
		NF	12		18 (r-l-ATT)	18		16 (l-OCC)	16
		13 (MOT)	13		NF	21		NF	21
		16 (DMN)	16						
		18 (l-OCC)	18						
		19 (MOT) *	19						
sub60624	22	3 (r-l-ATT)	3	18	3 (TEMP-MOT)	3	18	2 (TEMP-MOT)	2
		5 (FRO)	5		4 (DMN)	4		5 (l-OCC)	5
		6 (l-ATT)	6		5 (r-ATT)	5		6 (r-ATT)	6
		7 (TEMP-MOT)	7		7 (l-OCC)	7		8 (l-ATT)	8
		9 (r-ATT)	9		8 (l-ATT)	8		10 (DMN)	10
		10 (DMN)	10		9 (DMN)	9		13 (m-OCC)	13
		11 (DMN)	11		12 (m-OCC)	12		14 (DMN)	14
		17 (m-OCC)	17		NF	15		NF	17
sub76987	17	4 (r-l-ATT)	4	21	1 (l-OCC)	1	20	2 (TEMP-MOT)	2
		6 (DMN)	6		2 (EXC)	2		NF	5
		7 (m-OCC)	7		3 (DMN)	3		6 (DMN)	6
		9 (TEMP-MOT)	9		5 (l-ATT)	5		9 (m-OCC)	9
		10 (r-l-ATT)	10		6 (l-OCC)	6		10 (EXC)	10
		11 (EXC)	11		10 (m-OCC)	10		11 (r-l-ATT)	11
		12 (l-OCC)	12		11 (EXC)	11		12 (r-l-ATT)	12
					12 (MOT)	12			
					13 (TEMP-MOT)	13			
					15 (r-ATT)	15			

S	ICs	Visual Selection	M	ICs	Visual Selection	M	ICs	Visual Selection	M
sub84403	19	1 (FRO)	1	16	1 (m-OCC)	1	17	3 (DMN)	3
		NF	2		2 (TEMP-MOT)	2		5 (EXC) *	5
		3 (MOT)	3		5 (DMN)	5		NF	6
		6 (l-OCC)	6		9 (l-ATT)	9		10 (m-OCC)	NF
		8 (TEMP)	8		11 (r-l-ATT)	11		11 (DMN)	11
		NF	9		13 (EXC)	13		12 (TEMP-MOT)	12
		11 (r-l-ATT)	11		16 (l-OCC)	16		13 (l-OCC)	13
		14 (DMN)	14					14 (l-ATT)	14
		16 (m-OCC) *	NF					15 (r-l-ATT)	15
sub86146	21	4 (m-OCC)	4	19	4 (m-OCC)	4	16	7 (DMN)	7
		9 (EXC)	9		7 (DMN)	7		8 (r-l-ATT)	8
		10 (m-OCC)	10		9 (MOT)	9		9 (EXC)	9
		12 (DMN)	12		10 (EXC)	10		10 (DMN)	10
		14 (EXC)	14		12 (DMN)	12		13 (m-OCC)	13
		15 (l-ATT)	15		16 (r-l-ATT)	16			
		16 (r-l-ATT)	16						
		NF	17						
		19 (r-l-ATT)	19						
sub90179	27	NF	5	25	NF	2	24	NF	1
		7 (TEMP-MOT)	7		NF	3		2 (MOT)	2
		NF	11		NF	4		NF	4
		14 (DMN)	14		8 (EXC)	NF		9 (l-OCC)	9
		16 (m-OCC)	16		13 (l-OCC)	13		11 (TEMP-MOT)	11
		18 (r-l-ATT)	18		14 (DMN)	14		13 (r-ATT)	NF
		22 (MOT)	22		15 (r-l-ATT)	15		14 (DMN)	14
		24 (l-ATT)	24		16 (m-OCC)	16		15 (m-OCC)	15
					17 (MOT)	17		20 (EXC)	20
					19 (m-OCC)	19		22 (l-ATT)	22
					22 (r-l-ATT)	22		23 (DMN)	23
					24 (TEMP)	25		24 (l-OCC)	24
sub94293	26	1 (m-OCC)	1	20	2 (r-l-ATT)	2	22	2 (r-l-ATT)	2
		2 (r-l-ATT)	2		3 (TEMP-MOT)	3		4 (TEMP-MOT)	4
		4 (TEMP-MOT)	4		4 (l-OCC)	4		5 (r-l-ATT)	5
		7 (DMN)	7		5 (DMN)	5		6 (DMN)	6
		9 (DMN)	9		6 (DMN)	6		7 (DMN)	7
		10 (EXC)	10		8 (l-ATT)	8		8 (EXC)	8
		12 (l-ATT)	12		9 (FRO)	9		9 (r-l-ATT)	9
		13 (r-ATT)	13		10 (m-OCC)	10		10 (FRO)	10
		21 (MOT)	21		11 (MOT)	11		13 (TEMP-MOT)	13
		24 (MOT)	24		12 (DMN)	12		20 (m-OCC)	20

DMN = default-mode network, m-OCC (medial visual cortical areas), l-OCC (lateral visual cortical areas), r-ATT (right lateralized frontoparietal attention networks), l-ATT (left lateralized fronto-parietal attention networks), EXC (executive-control network), FRO (frontal network), TEMP (auditive system), MOT (sensorimotor cortex), TEMP-MOT (auditive system–sensirimotor cortex), NF = not found. **(*) the IC, evaluated discordantly by the two evaluators, is accepted after discussion within the group, (§) the IC, evaluated discordantly by the two evaluators, is rejected.**
